# Prevalence of parasitic infection in captive wild animals in Bir Moti Bagh mini zoo (Deer Park), Patiala, Punjab

**DOI:** 10.14202/vetworld.2016.540-543

**Published:** 2016-06-01

**Authors:** A. Q. Mir, K. Dua, L. D. Singla, S. Sharma, M. P. Singh

**Affiliations:** 1 Department of Veterinary Medicine, Guru Angad Dev Veterinary and Animal Sciences University, Ludhiana - 141 004, Punjab, India; 2 Department of Veterinary Parasitology, Guru Angad Dev Veterinary and Animal Sciences University, Ludhiana - 141 004, Punjab, India; 3 Mahendra Choudhury Zoological Park, Chhatbir, Punjab, India

**Keywords:** captive wild animals, carnivores, parasitic infection, Punjab, rodent, ungulates

## Abstract

**Aim:**

The study was conducted to know the prevalence of gastrointestinal parasites of captive wild animals at Bir Moti Bagh Mini Zoo (Deer Park), Patiala, Punjab.

**Materials and Methods:**

A total of 31 fecal samples from eight species of captive animals including Civet cat (*Viverra zibetha*), Porcupine (*Hystrix indica*), Nilgai (*Boselaphus tragocamelus*), Spotted deer (*Axis axis*), Black buck (*Antelope cervicapra*), Sambar deer (*Cervus unicolor*), Hog deer (*Axis porcinus*), and Barking deer (*Muntiacus muntjak*) were screened using classical parasitological techniques including sedimentation and floatation technique.

**Results:**

Out of 31 fecal samples examined, 20 were positive for parasitic ova/oocysts of different species indicating an overall prevalence of 68.0%. The six different types of parasites observed in the study included strongyle (67%), *Strongyloides* spp. (14%), coccidia (38%), *Trichuris* spp. (19%), ascarid (10%), and *Capillaria* spp. (10%). *Strongyles* were the most common parasites observed (67%) followed by coccidia (38%). Mixed helminth and protozoan infection were observed in 48% of animals. No cestode or trematodes were detected during the study.

**Conclusion:**

The high prevalence of gastrointestinal parasites without overt clinical signs of disease or mortality as observed in this study is suggestive of subclinical infection. The findings will help in formulating the appropriate deworming protocol for parasitic control in these captive animals.

## Introduction

Zoos are an *ex-situ* form of conservation where animals are displayed in cages or enclosures for esthetic, educational or research, and conservation purposes [1]. Zoo populations are distinctive as they are maintained to educate the public regarding wildlife and their habitats and/or to preserve critically endangered species through captive breeding and reintroduction programs [2]. Parasites and infectious diseases have become a major concern in conservation of endangered species as they can lead to mortality, dramatic population declines, and even contribute to local extinction events [3-6]. Some studies have revealed that gastrointestinal parasites of wild animals in captivity include zoonotic species to humans and raise public health concerns [7-12].

Parasitic diseases often represent a major concern in zoo animals for the high environmental contamination due to the maintenance of animals in confined areas [13,14]. In wild conditions, animals have some natural resistance against parasitic diseases and there is a state of equilibrium between the parasite and the host and it seldom lead to harmful infection unless stressed [15]. In captivity wild animals may succumb to parasitic infections due to environmental stress such as change in the living conditions and space limitations [16,17]. The constant stress of captivity makes animals more susceptible to parasitic infection as the immune system of these captive animals becomes weak [14,18]. Studies on parasitic diseases of wildlife are still in infancy in India with only few systematic studies having been undertaken, and data are still on the base line [19].

To have a better understanding about the prevalence of the endoparasites affecting zoo animals, this study attempts to investigate the occurrence of helminth parasites of animals in Bir Moti Bagh Mini Zoo (Deer Park), Patiala, Punjab.

## Materials and Methods

### Ethical approval

The ethics committee for animal experiments from the Guru Angad Dev Veterinary and Animal Sciences University granted an approval (IAEC/2014/1) during XXXIII meeting of Institutional Animal Ethics Committee for the conduction of work.

### Study site

The study was conducted at Bir Moti Bagh Mini Zoo (Deer Park) in the Patiala Wildlife Sanctuary located between 30°17’13.86”N latitude and 76°23’47.09”E longitude and at an altitude of 258 m (846 ft) during Jan-Feb, 2015. The sanctuary is located at a distance of 5 km from Patiala on Patiala-Dakala Road and is spread over 654 ha of state land. This mini zoo is home to nine species of mammals with 213 animals. The climate of the place is generally dry with rains during monsoon season. The average maximum and minimum temperature during January is 20.2°C and 7.1°C, respectively, while in June maximum temperature can go up to 44.4°C. The average annual rainfall in the district is 604.3 mm.

The zoo is separated into open enclosures for ungulates with different species having separate units except that of Black buck which is kept in two units. Civet cat and porcupines are housed in closed enclosures. The indoor and outdoor enclosures are cleaned on a routine basis with necessary prophylaxis. The prophylactic measures apart from regular deworming involved using the foot dips at the entrance of the zoo and also at each enclosure, tilling of enclosure area at regular intervals and adding some lime to destroy the larvae. The animals are fed black gram, cattle feed, and green fodder on daily basis with jaggery (gur) once a week. The zoo is a closed type having no introduction of any outside animal at least for past 5 years.

### Animals sampled

A total of 31 fecal samples from eight species of animals belonging to ungulates, felines, and rodents were collected (Table-1). Among the eight species of captive animals surveyed in this study, 1 (3.2%) was felid, 2 (6.4%) were rodent, and 28 (90%) were ungulates. Fresh samples were collected in polythene bags from individual enclosures housing different species of animals. The properly labeled interlocked polythene bags containing the fecal samples were brought to the laboratory of the Department of Parasitology, Guru Angad Dev Veterinary and Animal Sciences University, for parasitological examination.

**Table-1:** Prevalence of parasitic infection in captive wild animals in Bir Moti Bagh mini zoo.

Animals	Total animals	Sample collected	Positive (%)	Parasite observed						Mixed infection

				Nematodes					Protozoa	
	
				Strongyle	*Trichuris*	Ascarid	*Strongyloides*	*Capillaria*	Coccidia	
Civet cat (*V. zibetha*)	01	01	01 (100)	01	-	01	-	-	-	01^a^
Porcupine (*H. indica*)	05	02	02 (100)	-	02	01	-	02	-	02^b,c^
Nilgai (*B. tragocamelus*)	09	04	04 (100)	02	01	-	01	-	04	03^d,e^
Spotted deer (*A. axis*)	62	08	04 (50)	03	01	-	-	-	01	01^f^
Black buck (*A. cervicapra*)	98	08	06 (75)	06	-	-	01	-	01	02^e,g^
Sambar (*C. unicolor*)	10`	03	02 (66)	01	-	-	-	-	02	01^e^
Hog deer (*A. porcinus*)	26	04	01 (25)	01	-	-	-	-	-	-
Barking deer (*M. muntjak*)	02	01	01 (100)	-	-	-	01	-	-	-
Percent prevalence	213	31	21 (68)	14 (67%)	04 (19%)	02 (10%)	03 (14%)	02 (10%)	08 (38%)	10 (48%)

a Strongyle and *Toxascaris leonine,*

b *Trichuris*, *Capillaria* and ascarid,

c *Trichuris* and *Capillaria*,

d Coccidia, *Trichuris* and *Strongyloides*,

e Coccidia and Strongyle,

f *Trichuris* and Coccidia,

g Strongyle and *Strongyloides*. *V. zibetha=Viverra zibetha, H. indica=Hystrix indica, B. tragocamelus=Boselaphus tragocamelus, A. axis=Axis axis, A. cervicapra=Antelope cervicapra, C. unicolor=Cervus unicolor, A. porcinus=Axis porcinus, M. muntjak=Muntiacus muntjak*

### Sample processing

The fecal samples were subjected to detailed routine parasitological analysis for the presence of parasitic eggs/oocysts by direct smear examination, standard sedimentation, and floatation techniques [20].

## Results and Discussion

This study revealed the presence of both helminth and protozoan parasites in Bir Moti Bagh Mini Zoo (Deer Park), Patiala, Punjab. Out of the total 31 fecal samples screened, 21 (68%) samples were positive for gastrointestinal parasitic infection as given in Table-1. Previously, different workers have reported the prevalence of similar parasitic infection in captive zoo animals from Nigeria, Italy, and Malaysia and among workers and inmates in a Nigerian zoo [10-12,21,22]. However, the prevalence was higher than reported earlier by other authors [1,23,24]. The eggs/oocysts of six different types of parasites were observed in the study including strongyle (67%), *Strongyloides* spp. (14%), coccidia (38%), *Trichuris* spp. (19%), ascarid (10%), and *Capillaria* spp. (10%)*. Strongyles* spp. were the most common parasites observed (67%) followed by coccidia (38%). Infections with trematodes and cestodes were not detected in this study. Helminth infection was more common than protozoal infection with nematode eggs observed in 19 (90%) affected animals while protozoans were observed in only 8 (38%) animals out of the total positive animals [21]. Similar findings were reported by Fagiolini *et al*. and Lim *et al*. [21,22], who also reported higher helminth than protozoal infection in Italian zoo. However, it is contrary to the studies of Levecke *et al.*, Cordon *et al.*, and Gomez *et al*. [7,18,25], who reported higher protozoal infection compared to helminthic infection. 48% of the parasitic infections were mixed infections comprising two or more helminth or protozoan parasites, whereas 52% of the infections comprised of only one parasite. Among the ungulates, the highest prevalence of mixed infection was observed in Nilgai (75%). *Toxascaris leonina* was observed in captive civet cat in this study, and earlier studies have also reported the parasite from captive felids [26].

The eggs/oocysts of six different types of parasites, *viz*., strongyle, *Strongyloides* spp., coccidia, *Trichuris* spp., ascarid, and *Capillaria* spp. were observed in the study, and the majority of the animals examined in this study were infected with at least one intestinal parasite species. The gastrointestinal parasites species identified in this study have previously been recorded in captive animals in various zoos and zoological garden by other authors [27-29]. *Strongyles* were found to most prevalent parasites in this study which could be due to more conducive environment for the development of the pre-parasites stages in the hot and humid environmental conditions of this region [30,31].

The intensive husbandry of wild animals in zoos and zoological parks may be one of the reasons for the higher infection as high animal density in enclosures and their proximity to other species of animals provides opportunity for transmission of parasites [32]. Moreover, it has been observed that confinement of wild animals in zoo makes them more prone to different parasitic infections despite proper attention to feeding, water, and maintenance of hygiene in captivity [33]. The nematodes and some coccidian parasites have a direct life cycle, i.e., they do not involve any intermediate host and are transmitted by feco-oral route through contaminated feed, water, and soil and have the potential to accumulate in a captive environment [1]. Since all the parasites recorded in this study have direct life cycle and have the ability to survive in the environment, there is a high possibility of environmental contamination as the reason for their higher prevalence [7,18,25]. Trematodes and cestodes were not detected in this study. This could be due to the fact that these parasites (mainly trematodes and some cestodes) require an intermediate host for their transmission and are less likely to accumulate in the captive environment [17]. Since the wild animals in zoos are maintained in closed enclosures giving no chance of accessibility to the intermediate hosts of trematodes and cestodes. The environmental contamination could be through contaminated water or fodder, and even zoo workers have also been reported to play a role in transmission by acting as vectors and transmitting parasites through their shoes, clothes, hands, food, or with working tools [8,34,35]. In the present zoo, there was no foot dip at the entrance of each cage/enclosure although the foot dip was present at the entrance of the zoo there is possibility that zoo workers cleaning the cages and enclosures could act as a vehicle (fomite) for the transmission of parasites as the pathogens may be present in the zoo environs.

## Conclusion

As there was no reported mortality and clinical signs, and animal were apparently healthy during the period of examination, the high prevalence indicates subclinical infection which may flare up under stress conditions and can cause pathogenicity. Although overall management of zoo including nutrition, sanitation, and deworming practices was followed, the study identifies that there is scope for improvement in the management of the zoo by re-standardizing/re-investigating or re-scheduling anthelmintic program, regular examinations for parasitic infections and early season treatments to prevent infection.

## Authors’ Contributions

AQM performed the study and wrote the manuscript, KD and LDS helped in planning, execution of work, and edited the manuscript, SS helped in analysis, and MPS contributed for collection of samples from wild animals.
